# Supplementary material for: Multi-omics analysis identifies drivers of protein phosphorylation
Source: Genome Biol. 2023 Mar 21;24:52. doi: 10.1186/s13059-023-02892-2 (PMC10031968; doi:10.1186/s13059-023-02892-2)
Supplement: Supplementary file 1 — Additional file 1: Figure S1. Overview of protein and phosphopeptide quantification. Figure S2. Sex effects and heritability on the abundance of proteins and phosphopepitdes across tissues. Figure S3. pQTL and phQTL mapping from CC strains in heart, kidney and liver tissues. Figure S4. Mediation of phQTL through the abundance of their parent proteins (substrates). Figure S5. NZO allele drives the low abundances of MCAT pS41 in CC strains. Figure S6. pQTL and phQTL were identified to regulate phosphopeptide abundance together. Figure S7. Phosphorylation sites on one protein can be regulated coordinated and not coordinated. [file 13059_2023_2892_MOESM1_ESM.docx]

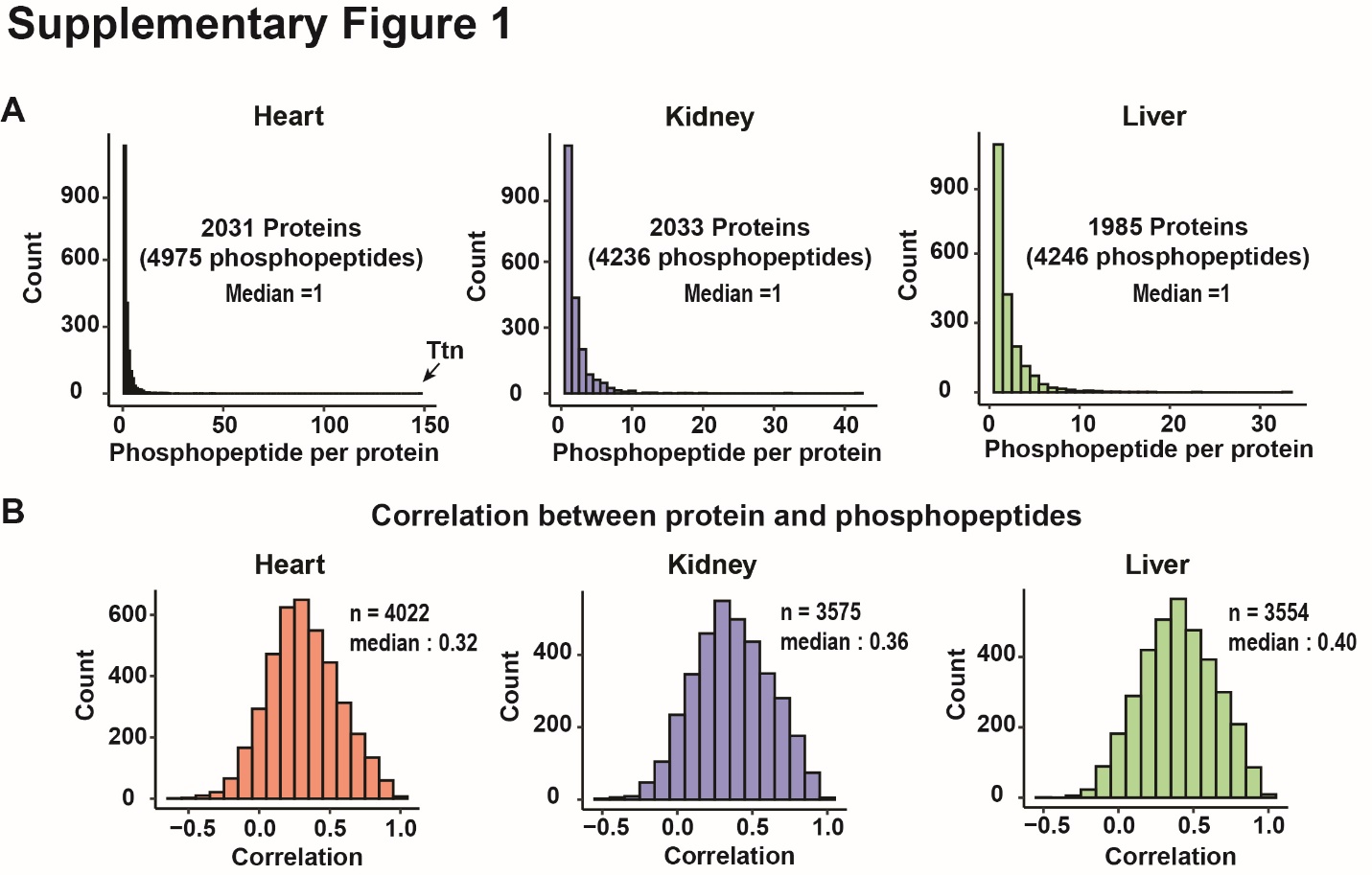


**Figure S1 (Related to Figure 1). Overview of protein and phosphopeptide quantification.** **(A)** Histogram of the number of quantified phosphorylation events per protein in three tissues. **(B)** Phosphopeptide abundances were highly correlated with their parent protein abundances in all three tissues. **(C)** Adjusted phosphopeptide abundances were not correlated with their parent protein abundances in all three tissues as expected.


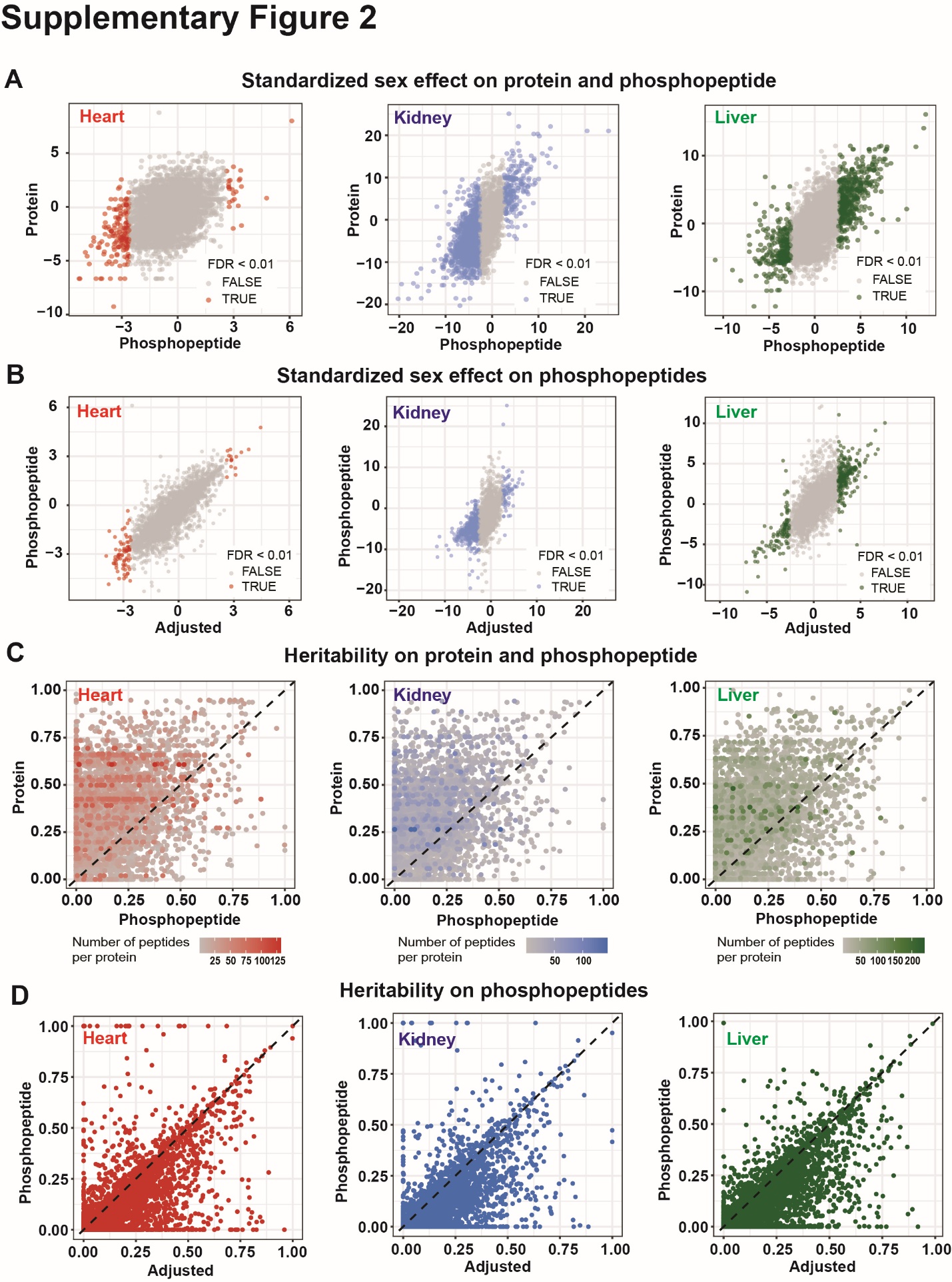


**Figure S2. Sex effects and heritability on the abundance of proteins and phosphopepitdes across tissues.** **(A)** Comparison of sex effects (difference/SE; female as reference) on phosphopeptide abundance and their parent protein abundance in heart, kidney and liver tissues. **(B)** Comparison of sex effect on phosphopeptide abundance before and after adjusting for parent protein abundance in heart, kidney and liver tissues. **(C)** Comparison of heritability on phosphopeptide abundance and their parent protein abundance in heart, kidney and liver tissues. **(D)** Comparison of heritability on phosphopeptide abundance and adjusted phosphopeptide abundance in heart, kidney and liver tissues.


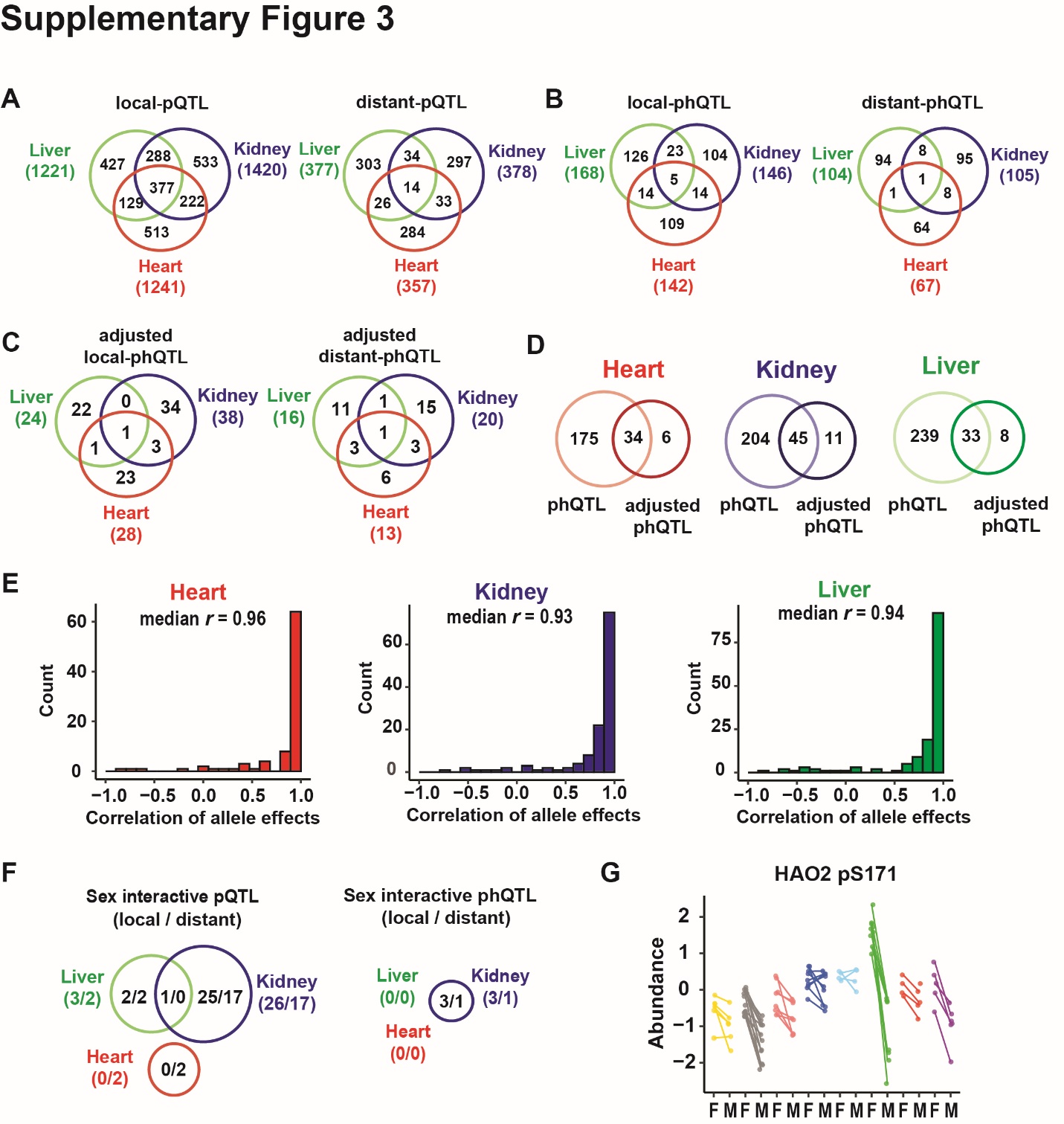


**Figure S3. pQTL and phQTL mapping from CC strains in heart, kidney and liver tissues**. **(A)** Venn diagram of the identified local pQTL and distant pQTL for proteins in liver, kidney and heart tissues. **(B)** Venn diagram of the identified phQTL for phosphopeptides in liver, kidney and heart tissue. **(C)** Venn diagram of the identified adjusted phQTL for phosphopeptides in liver, kidney and heart tissue. **(D)** Venn diagram of the identified phQTL and adjusted phQTL for phosphopeptides in liver, kidney and heart tissue, respectively. **(E)** The correlation of genetic effects for identified phQTL and pQTL in their parent proteins were high with exceptions in heart, kidney and liver tissues. **(F)** Venn diagram of detected sex-interactive pQTL and sex-interactive phQTL in three tissues. **(G)** HAO2 pS171 has a sex-interactive phQTL. Points are colored by founder haplotype at sex-interactive phQTL. Males and females from the same CC strain were connected by a line.


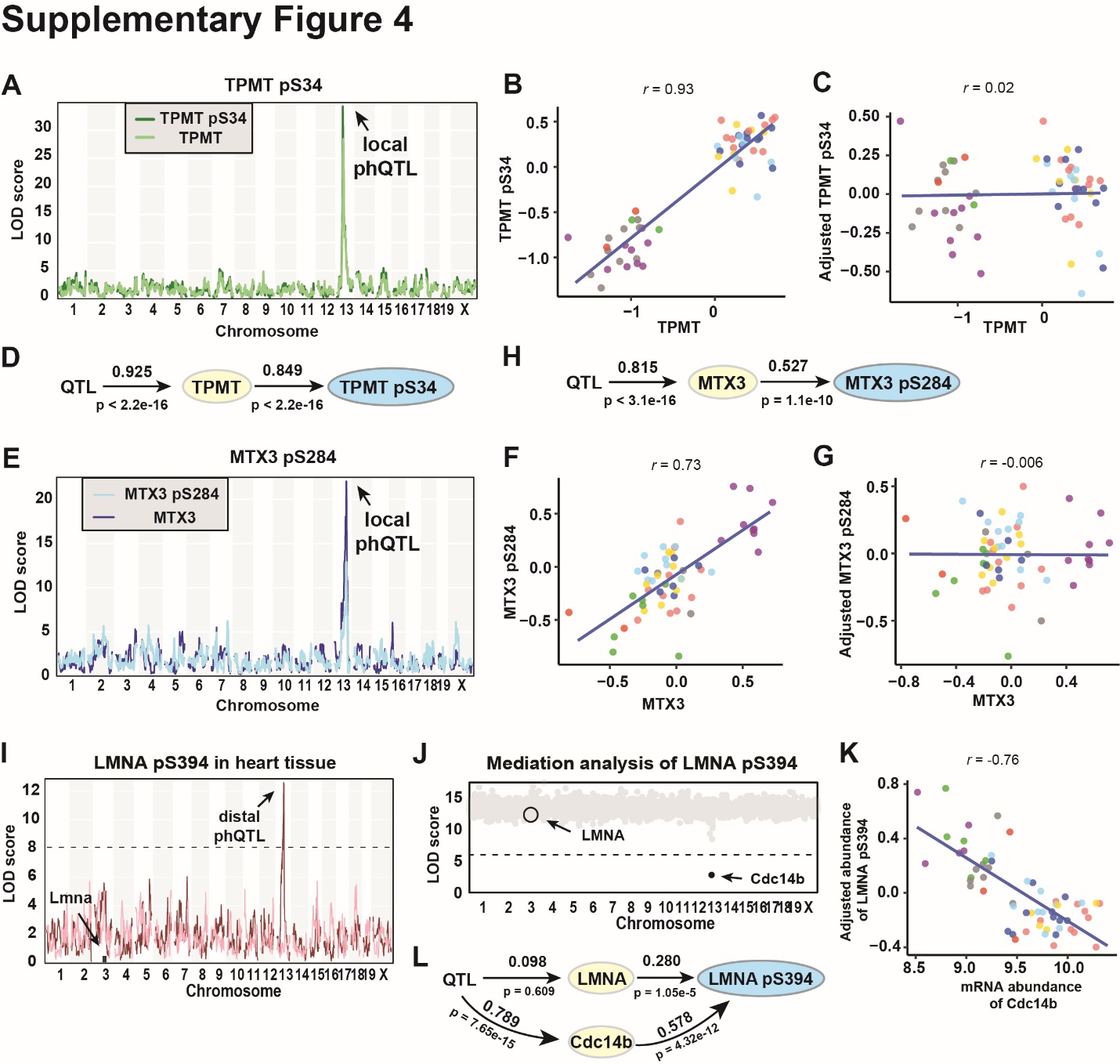


**Figure S4. Mediation of phQTL through the abundance of their parent proteins (substrates).** **(A)** Genome scans of TPMT and TPMT pS34 in liver tissue. **(B)** The abundances of TPMT and TPMT pS34 were highly correlated (*r* =0.93). **(C)** After adjusting for TPMT abundance, TPMT pS34 abundance is no longer correlated with TPMT abundance. Points are colored based on founder haplotype at *Tpmt*. **(D)** Path diagram of TPMT pS34 abundance regulation in liver tissue. **(E)** Genome scans of MTX3 and MTX3 pS284 in kidney tissue. **(F)** The abundances of MTX3 and MTX3 pS284 were highly correlated (*r* =0.73). **(G)** After adjusting for MTX3 abundance, MTX3 pS284 abundance is no longer correlated with MTX3 abundance. Points are colored based on founder haplotype at *Mtx3*. **(H)** Path diagram of MTX3 pS284 abundance regulation in liver tissue.


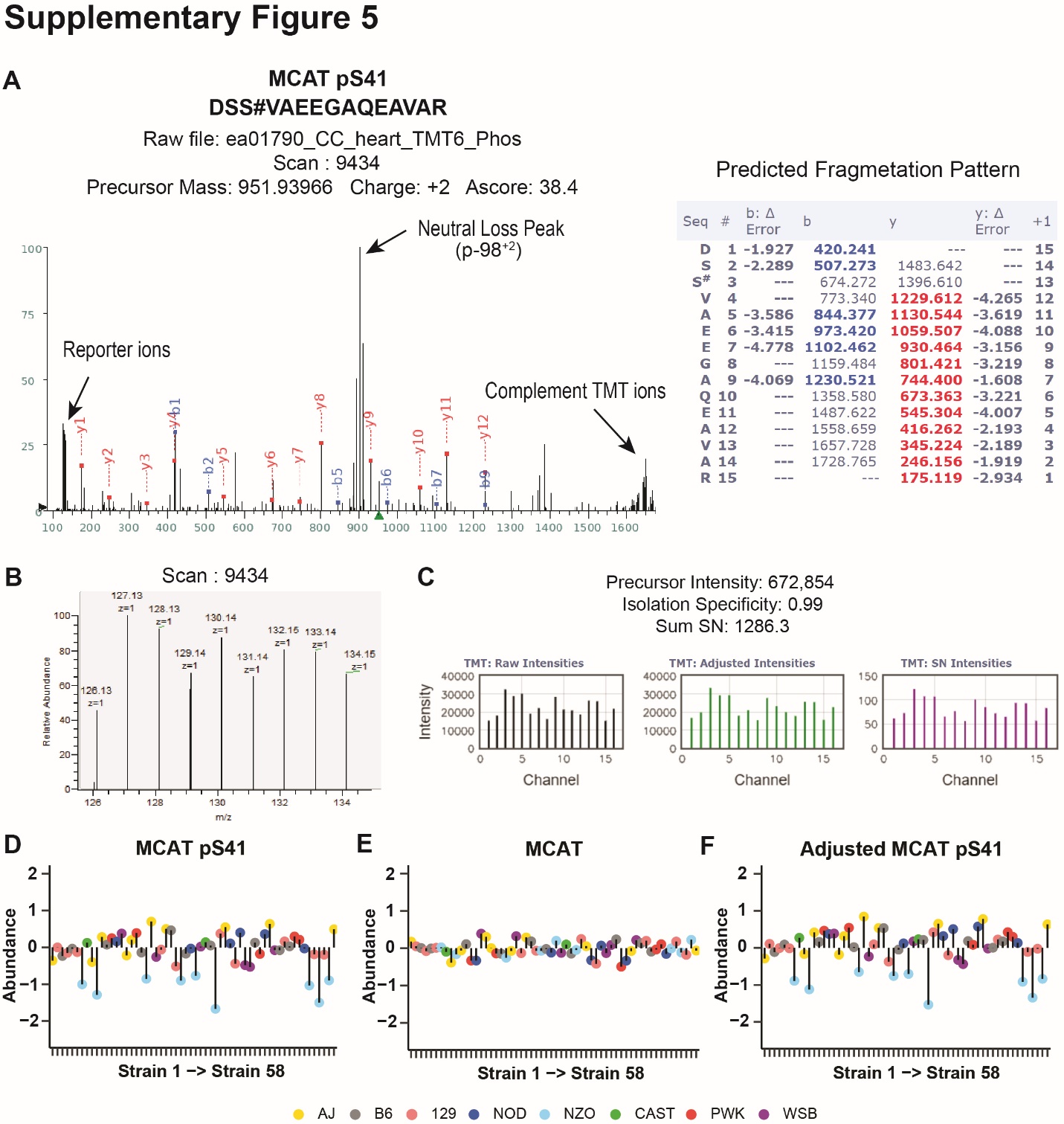


**Figure S5. *NZO* allele drives the low abundances of MCAT pS41 in CC strains.** **(A)** An example spectrum of identified phosphopeptide DSS#VAEEGAQEAVAR which harbors MCAT pS41. Match ions were highlighted in blue or red in the Predicted fragmentation table on the right. Reporter ions **(B)** of this spectrum was extracted and processed **(C)**. Signal to noise intensities were used for further analysis. **(D)**. *NZO* allele drives the low abundances of MCAT pS41 in 58 CC strains. Dots were colored based on the founder haplotye at the identified pQTL of MCAT pS41. **(E)** MCAT abundances have minimal variation in 58 CC strains. Dots were colored based on the founder haplotye at the identified pQTL of MCAT pS41. **(F)** *NZO* allele drives the low abundances of MCAT pS41 in 58 CC strains. Dots were colored based on the founder haplotye at the identified pQTL of MCAT pS41.


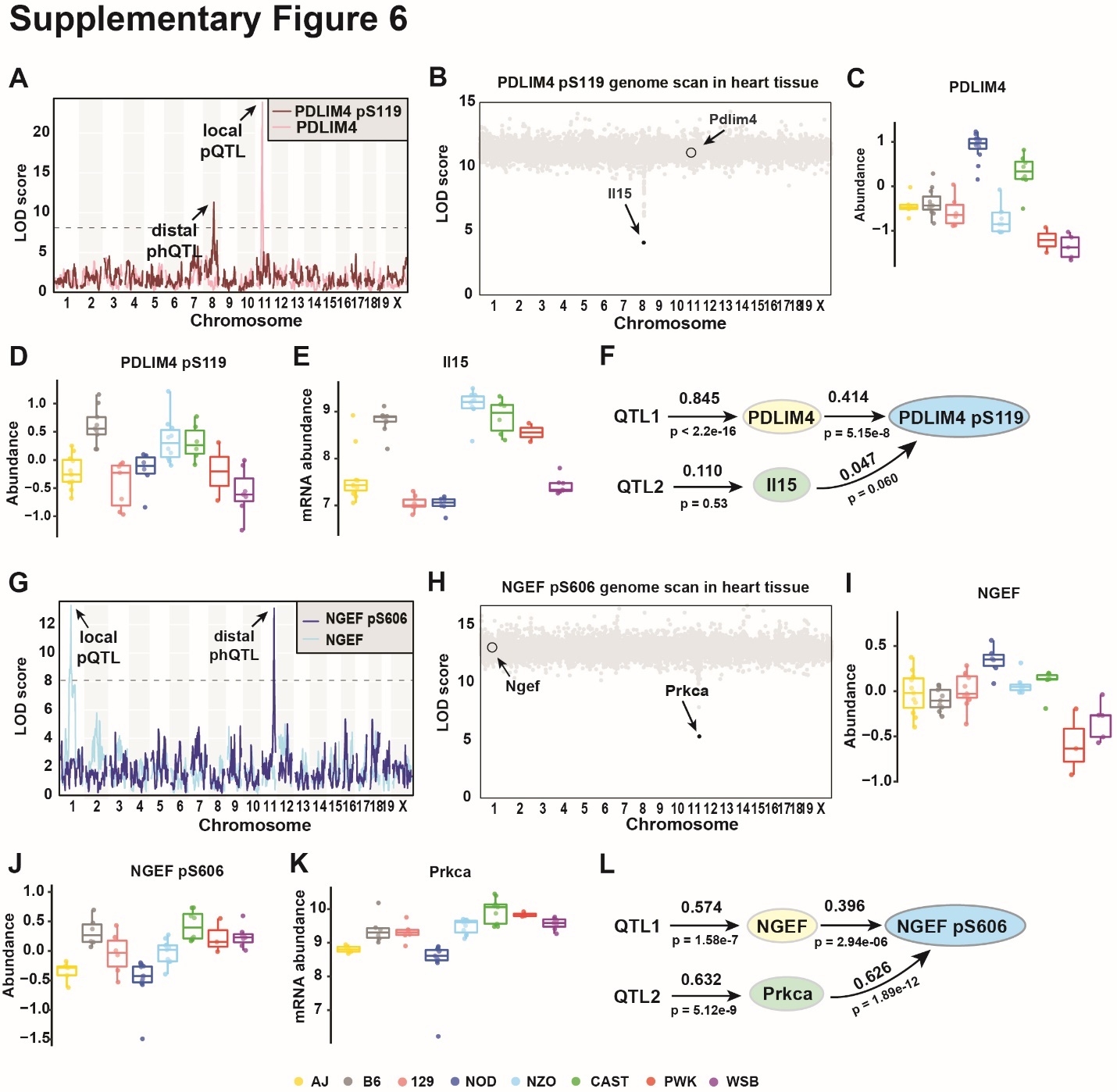


**Figure S6. pQTL and phQTL were identified to regulate phosphopeptide abundance together.** **(A)** Genome scans of PDLIM4 and PDLIM4 pS119 in heart tissue. **(B)** Mediation analysis using transcriptomics data identified *Il15* as the mediator for the phQTL of PDLIM4 pS119. Each gray dot is a mediation score representing the PDLIM4 pS119 LOD score conditioned on a transcript as candidate mediator. **(C)** The *NOD* and *CAST* alleles drove the high abundances of PDLIM4 in heart tissue. Data were categorized based on the founder haplotye at the identified pQTL. Abundances of (**D**) PDLIM4 pS119 and (**E**) *Il15* transcript had similar patterns based on the founder haplotype at *Il15*. **(F)** Path diagram of PDLIM4 pS119 abundance regulation in heart tissue. **(G)** Genome scans of NGEF and NGEF pS606 in kidney tissue. **(H)** Mediation analysis using transcriptomics data identified *Prkca* as the mediator for the phQTL of NGEF pS606. Each gray dot is a mediation score representing the NGEF pS606 LOD score conditioned on a transcript as candidate mediator. **(I)** The *PWK* and *WSB* alleles drove low abundance of NGEF in kidney tissue. Data were categorized based on the founder haplotye at the *Ngef*. Abundances of **(J)** NGEF pS606 and **(K)** *Prkca* transcripts had similar patterns. Data were categorized based on the founder haplotye at the *Prkca*. **(L)** Path diagram of NGEF pS606 abundance regulation in heart tissue.


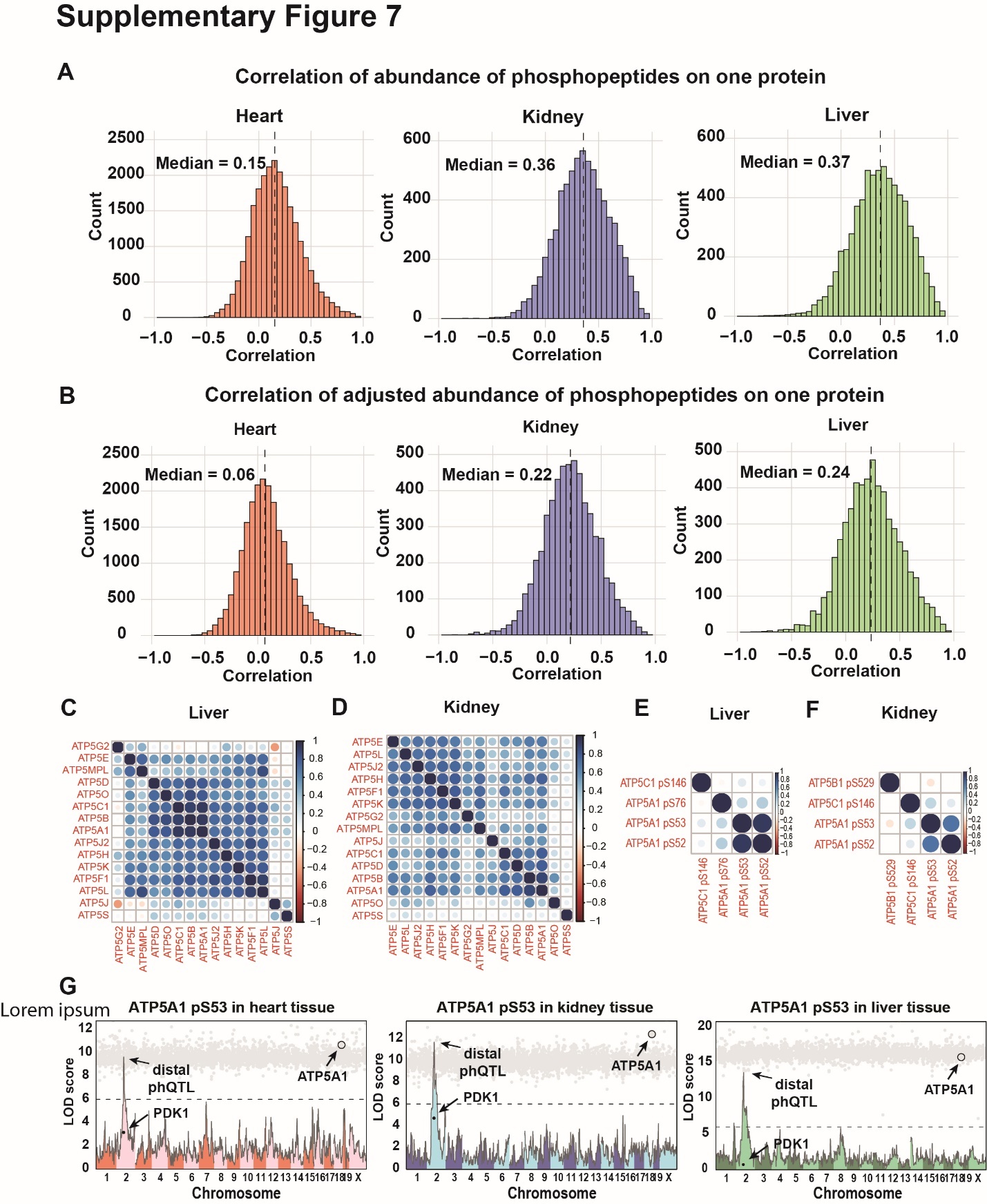


**Figure S7. Phosphorylation sites on one protein can be regulated coordinated and not coordinated. (A)** Correlation of the abundances of phosphopeptides from the same protein in three tissues. **(B)** Correlation of the adjusted abundances of phosphopeptides from the same protein in three tissues. Protein abundance of subunits in ATP synthase complex were highly correlated in the **(C)** liver and **(D)** kidney tissues. Phosphopeptide abundance from the ATP synthase complex were not as correlated in **(E)** liver tissue and **(F)** kidney tissue. **(G)** Genome scans of ATP5A1 pS53 overlayed with mediation scores in (left) heart, (middle) kidney and (right) liver tissues. Each gray dot is a mediation score representing the ATP5A1 pS53 phQTL LOD score conditioned on a protein as candidate mediator.
