# Supplementary material for: Multi-omics analysis identifies drivers of protein phosphorylation
Source: Genome Biol. 2023 Mar 21;24:52. doi: 10.1186/s13059-023-02892-2 (PMC10031968; doi:10.1186/s13059-023-02892-2)
Supplement: Supplementary file 7 — Additional file 7. Review history. [file 13059_2023_2892_MOESM7_ESM.docx]

Review History

**First round of review**

**Reviewer 1**

**Were you able to assess all statistics in the manuscript, including the appropriateness of statistical tests used?**

Yes, and I have assessed the statistics in my report.

**Comments to author:**

The authors have attempted to link genetic differences in mouse lines to quantified phosphorylation sites, to determine so called phQTLs, i.e. genetic regions that affect the normalised quantification of phosphorylation sites. Their method is relatively simple - they have quantified proteins and phosphopeptides via TMT from a large collection of mouse strains. Phosphopeptide abundance was normalised to remove changing effects of protein abundance. The linear mixed effect models were applied to link genetics differences to adjusted phosphopeptide abundance.

After data filtering and adjusting phosphopeptide abundance, around 1100 phosphopeptide quantified in 3 tissues, which is a relatively modest number. To achieve high coverage of phosphosites would have required deep fractionation, which I understand would have been exceptionally costly with so many mice strains to profile. Two tissue data sets were generated previously and a new tissue data set is reported here, generated on a different instrument - likely leading to some batch effects that will be difficult to remove. Despite having access to Orbitrap Eclipse and Lumos instruments, capable of collecting data at high-resolution, it appears that low-resolution MS3 spectra were collected, and even high-resolution MS1 and MS2 were searched with very broad tolerances of 50ppm. These settings will not assist with achieving confident PTM site localisation. Most problematically, no process was performed to run phosphosite location statistics, which means that the correct phosphosite will not be reported in many cases. Applying a rigorous site localisation approach, such as searching for a decoy phosphosite, such as pAla, would have allowed the investigators to determine how many false sites were being reported, and could indeed have used an approach with their decoy peptides and/or decoy phosphosites to determine the overall false discovery rate for phQTLs. Without these controls, I suspect that many spurious connections will be made

Furthermore, due to the normalisation mode employed, whereby protein abundance corrects phosphopeptide abundance, any incorrectly identified phosphopeptides will be more likely to have a completely difference abundant profile to the (false) parent protein, leading to apparent significance of the phosphopeptide. This problem is apparent in one of the key examples presented in Figure 2. A phosphopeptide apparently reporting pS272 is reported as being down in abundance female -> male, whereas the protein shows a slight increase female -> male, thus having a strong sex effect on the phosphosite. I searched through all the supplementary data to see if I could locate the evidence supporting this site's correct identification and quantification, but none seems to have been provided (apologies if missed it). By looking in public databases from which many mouse MS phosphoproteomics studies have been accumulated, there is no evidence that this is a phopshosite, and there are no nearby phosphosites that could have been confused (nor any in the orthologous region of rat or human). While it remains a possibility that the authors have discovered a new phosphosite, never previously observed, it is much more likely that insufficient statistical control somewhere in the pipeline has led to a false identification here - meaning that the apparent phQTL is incorrect.

Figure 4 (top panel) shows a possible phQTL, but here as noted by the authors, there is only protein abundance regulation by alleles, and not actual differences in the phosphosite abundance, once adjusted. The bottom panel and logic is pretty hard to follow. It is not helped since the axes on quantitative plots do not have a unit or explanation, log scales? The legends do not describe the data well, so I cannot tell whether differential quantification of the phosphosite is claimed or not.

Overall, while I think the concept is a good one - I don't see a compelling case in any figures presented, which carefully tracks through from protein abundance to phosphosite abundance (with an annotated spectrum to demonstrate site localisation is correct and that TMT quant has been performed correctly), showing that there is differential regulation of phosphosite abundance linked to a QTL. I am certain that there are alleles that affect protein abundance, and I also believe that the authors have identified some phosphosites on those proteins, but I do not see sufficient evidence in the publication (and supplements) that genetic differences have been linked to quantitative changes in site occupancy. For a new submission (here or elsewhere), the authors must provide all proteomics data as a supplement - quantification tables for all proteins, and all phosphopeptides (with spectrum identifiers to link back to raw data), and the results of adjustment. Data are presented in such a post-processed format that it is impossible to track back to see which connections might be real and which spurious/false positives.

**Reviewer 2**

**Were you able to assess all statistics in the manuscript, including the appropriateness of statistical tests used?**

Yes, and I have assessed the statistics in my report.

**Comments to author:**

In Zhang et al, the authors report on one of the most comprehensive assessments of the extent to which phosphorylation activities are genetically regulated in a mammalian system. The paper is well written, data is state-of-the-art, and overall, the authors provide a useful framework heavily based on mediation analysis in order to dissect the drivers of phosphorylation regulation , independent of contributions from protein levels alone.

It would be interesting to place the study's results in the context of the entire set of estimated kinase-target relationships. How many gene-phospho pairs are there observed with a similar correlation level, that is not genetically regulated.

No description of the HPLC conditions and fractionation schema for the basic pH HPLC analysis (see Page 14).

This statement is confusing "Previously for liver data, we only filtered out polymorphic

peptides with intensity data that approximately matched the expected B6-allele-to-strain pattern from the founder strain genome sequences, but here remove them all to avoid the possibility a peptide would be filtered from one tissue but retained in another".

It is not clear if phosphopeptides that harbor a polymorphism would be appropriate to compare across mouse strains, since the ionization efficiency of the peptides may differ, even from a single changes of an amino acid. I think the authors should discuss this limitation (see Page 18, Line 13-19). Also, the filtering criteria is not clear. Do the authors mean three AAs flanking the phospho-site, or the polymorphism from CC?

Figure S2C is not called out in the text.

The cases in which an adj-phQTL is found but not a cognate phQTL is very interesting. Can the authors comment on these cases.

How many polymorphic peptides were removed, compared to the total (see Page 17-18)?

Based on the sampling sensitivity of phospho-proteomics, a certain fraction of the phosphorylation sites would be assayed. Can the authors provide estimates, based on their knowledge of MS and genetics how many phosphorylation events may be genetically regulated?

Supplementary Figure 1A - Why are there fewer phosphorylation sites, on average, for heart?

Supplementary figure 1C is missing, and is not called out in the text.

How the standardized sex effect is computed is not entirely clear. I'd suggest the authors provide a bit more methodological information describing how the histograms in Figure 2A are generated.

\### Minor comments:

Page 5 Line 29 - should be "pQTL" here? "distant phQTL (11% are present in at least two tissues)"

Page 3 Line 7-8 "i.e.,"

Page 3 Lines 45-46 Would say only slightly correlated, not "correlated", given the R-squared values

Page 32 Line 4 "(middle), and"

Seems like phQTL is being used for phenotype QTL

**Authors Response**

**Point-by-point responses to the reviewers’ comments:**

Response to Reviewers

*Reviewer #1: The authors have attempted to link genetic differences in mouse lines to quantified phosphorylation sites, to determine so called phQTLs, i.e. genetic regions that affect the normalised quantification of phosphorylation sites. Their method is relatively simple - they have quantified proteins and phosphopeptides via TMT from a large collection of mouse strains. Phosphopeptide abundance was normalised to remove changing effects of protein abundance. The linear mixed effect models were applied to link genetics differences to adjusted phosphopeptide abundance.*
 *After data filtering and adjusting phosphopeptide abundance, around 1100 phosphopeptide quantified in 3 tissues, which is a relatively modest number. To achieve high coverage of phosphosites would have required deep fractionation, which I understand would have been exceptionally costly with so many mice strains to profile.*

*Reply: We wholeheartedly agree that performing phosphoproteomics screens at this scale is a nontrivial undertaking, and the quality of the data will depend on many factors, including how these data were acquired on the instruments and how we analyzed it. Thus, we have paid close attention to these issues and believe that our methods are sound for several reasons.*

1. *The reviewer is correct that it would have required up to 10x the work and cost to perform deep phosphoproteome profiling across this large cohort of mice. Our phosphorylation method has the bonus that it is carried out on the samples as the protein measurements. In addition, because* *the labeling occurs prior to the enrichment, some of the error due to variability in the IMAC enrichment is removed.*
2. *In our analysis, we only used peptides with TMT reporter summed signal-to-noise of greater than 100 and quantified in at least six out of twelve TMT experiment in liver tissue and four out of eight TMT experiments in heart and kidney tissues.*
3. *We pre-adjusted for the effect of TMT batch using a linear mixed effect model (LMM) prior to QTL mapping.*
4. *Many of the seminal papers in this field (site localization and phosphorylation analysis) have been generated in the Gygi lab. Our localization software, AScore ^1^, was the first paper to directly address the localization issue through a probability score. We evaluate site localization by running AScore in this study.*

Two tissue data sets were generated previously, and a new tissue data set is reported here, generated on a different instrument - likely leading to some batch effects that will be difficult to remove. Despite having access to Orbitrap Eclipse and Lumos instruments, capable of collecting data at high-resolution, it appears that low-resolution MS3 spectra were collected, and even high-resolution MS1 and MS2 were searched with very broad tolerances of 50ppm. These settings will not assist with achieving confident PTM site localisation.

*Reply: To be clear, when collecting MS3 spectra, they are always collected in the orbitrap (high resolution). This is required to quantify the near-isobaric reporter ions (e.g., 127N and 127C).*

*Peptide precursor tolerance was set as we usually do to 50 ppm (MS1 scans). We have found that this wider window allows the software to better classify spectra as true or false positives. ^1^ There is a tradeoff due to losing a few low scoring MS/MS that are true positives, but you recover even more true positives due to the wider window.*

Most problematically, no process was performed to run phosphosite location statistics, which means that the correct phosphosite will not be reported in many cases. Applying a rigorous site localisation approach, such as searching for a decoy phosphosite, such as pAla, would have allowed the investigators to determine how many false sites were being reported, and could indeed have used an approach with their decoy peptides and/or decoy phosphosites to determine the overall false discovery rate for phQTLs. Without these controls, I suspect that many spurious connections will be made.

*Reply: It is true that identification of phosphorylation sites in phosphoproteome data can be difficult, especially across large number of samples, and if the data are handled improperly, there is the potential for false positives to accumulate. However, we are confident in the phosphorylation sites we have identified and the resulting correlations we have derived due to our multi-level approach for filtering data as described below.*

*There are two ways false positive sites arise: false positive peptide identifications and mislocalizations. We have addressed both of these using established techniques.*

*We ran the AScore algorithm to evaluate mislocalization ^1^ and* *provide an estimate for the probability of correct localization for each phosphorylation site. Specifically, a threshold of 20 corresponded to 99% confidence in site localization, while a threshold of 13 corresponded to 95% confidence in site localization.* *84%, 89% and 90% of the quantified phosphopetide used for analysis have an AScore greater than 13 in heart, liver, kidney tissues, respectively (P<0.05). All the information were uploaded to figshare (https://figshare.com/projects/Multi-omics_analysis_identifies_drivers_of_protein_phosphorylation/137673, Under the folder titled “Raw summary of protein and phosphopeptides quantitation” - siteQuant5100.csv). AScore of each phosphopeptide quantified were reported (Shown as “Max Score”). We have added this information to the manuscript in “Mass spectrometry data analysis” under Methods section.*

*We addressed false IDs by rigorous filtering at the phosphopeptide level (<1% FP allowed) and then by combining the phosphopeptides into phosphoproteins, which additionally filtered to less than 1% FDR at the protein level. Furthermore, we required a site to appear in at least half of the samples to include it in our QTL analyses. This means that we often had more than a dozen examples of a peptide being identified, and the site being localized, which adds confidence in the data quality.*

*While we have taken these steps to minimize false positive detection of phosphorylation sites, the QTL mapping results also provide strong support for the presence of a phosphorylation site. Detection of a QTL for a false positive phosphopeptide would require a highly unlikely chance association between genotype and phosphopeptide abundance to map a QTL that exceeds the stringent multiple test adjusted threshold for QTL detection. In summary, there are multiple steps to prevent false positive phQTL detection. We have high confidence in the reported phosphorylation events with QTL due to the stringency of their detection and identification through mass-spec, and a high threshold for declaring significant genetic associations.*

Furthermore, due to the normalisation mode employed, whereby protein abundance corrects phosphopeptide abundance, any incorrectly identified phosphopeptides will be more likely to have a completely difference abundant profile to the (false) parent protein, leading to apparent significance of the phosphopeptide.

*Reply: Since most sites and most proteins show relatively few changes, this is not necessarily true. It would be a rare event if a false positive phosphopeptide was identified over and over again in at least half the samples and was assigned to a protein that was quantified in the dataset, and that protein had a changing expression profile. The FP are at less than 1% across the dataset prior to enforcing the “found in half” requirement which would remove nearly all FP. As we mentioned before, this false adjusted phosphopeptide would have to by chance associate with genotype (at a level beyond our QTL significance thresholds), making it very unlikely that we report false positive adjusted phQTL.*

This problem is apparent in one of the key examples presented in Figure 2. A phosphopeptide apparently reporting pS272 is reported as being down in abundance female -> male, whereas the protein shows a slight increase female -> male, thus having a strong sex effect on the phosphosite. I searched through all the supplementary data to see if I could locate the evidence supporting this site's correct identification and quantification, but none seems to have been provided (apologies if missed it). By looking in public databases from which many mouse MS phosphoproteomics studies have been accumulated, there is no evidence that this is a phopshosite, and there are no nearby phosphosites that could have been confused (nor any in the orthologous region of rat or human). While it remains a possibility that the authors have discovered a new phosphosite, never previously observed, it is much more likely that insufficient statistical control somewhere in the pipeline has led to a false identification here - meaning that the apparent phQTL is incorrect.

*Reply: In this case, close examination of the data makes us quite confident in its identification and quantification for several reasons.*

1. *When viewed individually, the PSMs supporting this site identification are high quality (see below). Excellent coverage of b- and y-type ions is observed throughout the length of the sequence. Moreover, since there is only a single phosphorylatable residue on this peptide, mislocalization is not possible.*

*Below is a figure showing an example spectrum of CGREF1 pS272. We successfully identified and matched the predicted fragments from the peptide GQAES#EGDVPGPR, which led to a high AScore (100% confidence and no other phosphorylateable residue).*

2. *We identified this peptide 18 times across the kidney data, allowing us to quantify its abundance in dozens of mice. Since false positive peptide identifications tend to occur at random, it is highly unlikely we would observe the same false positive peptide ID 18 times. Thus, this identification is highly confident.*
3. *We searched our data against the Ensembl database. Below we show the alignment of CGREF1 in the Uniprot and Ensembl databases. We queried the sequence from Ensembl, and Sbjct is the sequence from Uniprot. S228 in Uniprot is S272 in Ensembl.*

CGREF1 pS228 has been identified three times as a phosphosite. (https://www.phosphosite.org/proteinAction.action?id=4853399&showAllSites=true)

Figure 4 (top panel) shows a possible phQTL, but here as noted by the authors, there is only protein abundance regulation by alleles, and not actual differences in the phosphosite abundance, once adjusted. The bottom panel and logic is pretty hard to follow. It is not helped since the axes on quantitative plots do not have a unit or explanation, log scales? The legends do not describe the data well, so I cannot tell whether differential quantification of the phosphosite is claimed or not.

*Reply: This is precisely the point of this figure—that some phosphosites differ in abundance due to changes in their parent protein level and not due to differences in stoichiometry of the site. Phosphopeptide abundance can be driven by abundance of the parent protein (Mechanism 1) and by factors that affect phosphorylation levels independently of protein abundance (Mechanism 2; Fig. 4A). In the top panel, GAS2 pS283 was shown as an example regulated only through its parent protein (Mechanism 1), while in the bottom panel, MCAT pS41 was shown as an example which is regulated independently of MCAT. The location of the genetic variant regulating MCAT pS41 is on chromosome 2 while Mcat locates at chromosome 15 (Fig. 4H). We performed mediation analysis (Methods Section) and identified PDK1 as the upstream regulator of MCAT pS41 abundances. The abundance of PDK1 and the adjusted abundance of MCAT pS41 are highly correlated (r= 0.86) (Fig. 4M). Furthermore, we identified additional phosphopeptides that were regulated through PDK1 (Fig. 4N).*

*In Figure 4D, phosphopeptide abundances were grouped based on the genotype of the local phQTL on chromosome 7, and PWK allele drove the low abundance of GAS2 pS283 through GAS2 protein. In Figure 4I, phosphopeptide abundances were grouped based on the genotype of the identified distant phQTL on chromosome 2, and NZO allele drove the low abundance of MCAT pS41. In Figure 4L, PDK1 abundances* *were grouped based on the genotype of the distant phQTL with MCAT pS41 (same locus with Figure 4I). The pattern of MCAT pS41 and PDK1 is similar, and the abundances are correlated, indicating that NZO allele drove the low abundance of MCAT pS41 through PDK1 abundance.*

*We thank the reviewer for pointing out concerns about the axis scales. In reference to proteins and phosphopeptides, there are three distinct scales and we have revised the figures to be consistent in axis labeling as well as added explanatory text to the figure legends. “Abundance” is the batch adjusted log2 intensity of the mass spec quantitation. The batch adjustment results in a zero-centered scale but changes of 1 unit on this scale correspond to a 2-fold change. “Adjusted abundance” - these are residuals from regression of the phosphopetide adundance on the parent protein abundance. Again, these are zero centered and a unit change corresponds to a 2-fold difference. “Standardized effects”, as in Figures 2 and S2, are regression coefficients divided by their standard error. These can be interpreted as z-scores (Wald test) and do not correspond to a fold-change. In addition, the is an “Abundance” scale for mRNA which are reported from the variance stabilizing transformation (vst) in DESeq2. Unit differences in the vst scale correspond to a 2-fold change.*

*We updated the legend and attach the current legend here:*

***Figure 4. Phosphopeptide abundance can be regulated by substrate abundance dependent or non-substrate abundance dependent mechanisms.*** ***…*** ***(I)*** *NZO alleles at Pkd1 drove the low abundances of MCAT pS41 in heart tissue. Colors denote the founder haplotype of additive allele effects at the identified pQTL of MCAT pS41.* ***(J)*** *Mediation analysis identified PDK1 expression as the mediator of MCAT pS41 abundances. Each gray dot is a mediation score representing the MCAT pQTL LOD score conditioned on a protein as candidate mediator.* ***(K)*** *Path diagram of MCAT pS41 abundance regulation in heart tissue.* ***(L)*** *NZO alleles at Pkd1 drove the low abundances of PDK1 in heart tissue. Colors denote the founder haplotype of additive allele effects at the identified pQTL of MCAT pS41.* ***(M)*** *The adjusted abundances of MCAT pS41 and PDK1 were highly correlated (r = 0.86) in heart tissue.* ***(N)*** *Mediation analysis identified PDK1 as the mediator of several phQTL in heart, kidney and liver tissue, respectively.*

Overall, while I think the concept is a good one - I don't see a compelling case in any figures presented, which carefully tracks through from protein abundance to phosphosite abundance (with an annotated spectrum to demonstrate site localisation is correct and that TMT quant has been performed correctly), showing that there is differential regulation of phosphosite abundance linked to a QTL. I am certain that there are alleles that affect protein abundance, and I also believe that the authors have identified some phosphosites on those proteins, but I do not see sufficient evidence in the publication (and supplements) that genetic differences have been linked to quantitative changes in site occupancy.

*Reply: As requested by the reviewer, we have added a supplemental figure using MCAT pS41 as an example to show the annotated spectra and quantification. We note that the abundance of MCAT pS41 is not regulated through MCAT protein abundance.*

***Figure S5. NZO allele drives the low abundances of MCAT pS41 in CC strains.*** ***(A)*** *An example spectrum of identified phosphopeptide DSS#VAEEGAQEAVAR which harbors MCAT pS41. Match ions were highlighted in blue or red in the Predicted fragmentation table on the right. Reporter ions* ***(B)*** *of this spectrum was extracted and processed* ***(C)****. Signal to noise intensities were used for further analysis.* ***(D)****. NZO allele drives the low abundances of MCAT pS41 in 58 CC strains. Dots were colored based on the founder haplotye at the identified pQTL of MCAT pS41.* ***(E)*** *MCAT abundances have minimal variation in 58 CC strains. Dots were colored based on the founder haplotye at the identified pQTL of MCAT pS41.* ***(F)*** *NZO allele drives the low abundances of MCAT pS41 in 58 CC strains. Dots were colored based on the founder haplotye at the identified pQTL of MCAT pS41.*

For a new submission (here or elsewhere), the authors must provide all proteomics data as a supplement - quantification tables for all proteins, and all phosphopeptides (with spectrum identifiers to link back to raw data), and the results of adjustment. Data are presented in such a post-processed format that it is impossible to track back to see which connections might be real and which spurious/false positives.

*Reply: We have uploaded the data in Figshare (https://figshare.com/projects/Multi-omics_analysis_identifies_drivers_of_protein_phosphorylation/137673). The folder titled “Raw summary of protein and phosphopeptides quantification” includes all the quantitation information for all proteins and all phosphopeptides to link back to the raw data (PRIDE dataset PXD032843). Processed protein and phosphopeptide quantification were uploaded to the folder titled “Processed datasets”. We also provide extra information for data processing and scripts to generate all the figures in other folders under the project “Multi-omics analysis identifies drivers of protein phosphorylation” in Figshare (https://figshare.com/projects/Multi-omics_analysis_identifies_drivers_of_protein_phosphorylation/137673).*

Reviewer #2

In Zhang et al, the authors report on one of the most comprehensive assessments of the extent to which phosphorylation activities are genetically regulated in a mammalian system. The paper is well written, data is state-of-the-art, and overall, the authors provide a useful framework heavily based on mediation analysis in order to dissect the drivers of phosphorylation regulation, independent of contributions from protein levels alone.

It would be interesting to place the study's results in the context of the entire set of estimated kinase-target relationships. How many gene-phospho pairs are there observed with a similar correlation level, that is not genetically regulated.

*Reply: We thank the reviewer for the positive feedback. Phosphopeptide abundances are influenced by many factors, including substrate abundance, kinase abundance, kinase activity, phosphatase abundance, phosphopeptides activity and more. Here, we aim to identify how the genetic variation influences phosphopeptide abundances and furthermore, to identify candidate gene mediators of these genetic effects. In Figure 4H-N, we showed examples that a QTL in the genome controlled the abundance of PDK1 and the abundance of several phosphopeptides downstream of PDK1. In Table S5, we provided the list of protein and transcript mediators of phosphopeptide abundance, including 6 kinases, 3 phosphatases. There are many (thousands) of known kinase-substrate relationships and examining them all would be a daunting task. Moreover, we would not expect our data to be informative for the majority of these. In order to observe the correlation that indicates a causal relationship, there must be variation in the kinase (or other effectors). The genetic variants segregating in this population drive this variation and make the mediation analysis possible. In the absence of genetic variation, the observable correlations will be too weak to support the detection of a causal link.*

No description of the HPLC conditions and fractionation schema for the basic pH HPLC analysis (see Page 14).

*Reply: We fractionated the pooled TMT-labeled peptide sample using basic reverse-phase HPLC as described in the reference.*

*We used an Agilent 1260 pump equipped with a degasser and a single wavelength detector (set at 220 nm). Peptides were subjected to a 50 min linear gradient from 8% to 40% acetonitrile in 10 mM ammonium bicarbonate pH 8 at a flow rate of 0.6 mL/min over an Agilent 300Extend C18 column (3.5 μm particles,* *4.6 mm ID and 250 mm in length). The peptide mixture was fractionated into a total of 96 fractions which were consolidated into 24. Twelve fractions were desalted and analyzed by liquid chromatography-tandem mass spectrometry (LC-MS/MS).*

*We have added the above information to the manuscript in “Sample preparation for proteomics and phosphoproteomics analysis” under Methods section.*

This statement is confusing "Previously for liver data, we only filtered out polymorphic
peptides with intensity data that approximately matched the expected B6-allele-to-strain pattern from the founder strain genome sequences, but here remove them all to avoid the possibility a peptide would be filtered from one tissue but retained in another".

*Reply: We agree that this is confusing and have rephrased: “**We filtered out peptides with known polymorphisms in the 8 founder strains of the CC. This is a more stringent filter than was applied in our previous study of the liver data ^2^ and thus there are minor differences in the protein quantification.”*

It is not clear if phosphopeptides that harbor a polymorphism would be appropriate to compare across mouse strains, since the ionization efficiency of the peptides may differ, even from a single changes of an amino acid. I think the authors should discuss this limitation (see Page 18, Line 13-19). Also, the filtering criteria is not clear. Do the authors mean three AAs flanking the phospho-site, or the polymorphism from CC?

*Reply: As the reviewer notes, peptides harboring polymorphisms represent difficult cases, and, thus they were removed from the dataset if they were not 100% consistent (as tryptic peptides) across the 8 founder genomes. For phosphopeptide analysis, we also confirmed that three amino acids before and after the phosphopeptide were the same across all strains to avoid potential influence on trypsin cleavage.*

*For example, phosphopeptide K.TS#PVQSK.S from GAS2 protein. We confirmed that the peptide TSPVQSK is shared across all eight founder strains. Then, we confirmed that this peptide with three flanking amino acids DGKTSPVQSKSPT is also shared across eight founder strains. Therefore, the QTL scan was performed on this phosphopeptide.*

Figure S2C is not called out in the text.

*Reply: We have added this to the manuscript in “Heritability of phosphopeptides” under Results section.*

The cases in which an adj-phQTL is found but not a cognate phQTL is very interesting. Can the authors comment on these cases.

*Reply: In most cases where an adj-phQTL is found but not the cognate phQTL, this is due to the phQTL failing to meet stringent genome-wide significance. The process of computing the adjusted abundance of a phosphopeptide can reduce the biological error variation, which will increase the power for QTL detection. Below is an example.*

A peptide from gene Nucb1 provides an example of an adj-phQTL that does not have a corresponding phQTL. The adj-phQTL LOD (10.8) exceeds our significance threshold. There is a clear peak in the phQTL scan but LOD (6.9) is below the threshold for significance. The allele effects for the adj-phQTL and the phQTL are the same, NZO high & CAST low, indicating that these are very likely to be the same genetic effect. The gene encoding Nucb1 is on chr 7 and the QTL is on chr 11, so this a distal QTL mediated by mechanism 2 but no clear candidate mediator. Images from <https://churchilllab.jax.org/qtlviewer/cc_phospho_peptides>.

How many polymorphic peptides were removed, compared to the total (see Page 17-18)?

*Reply: Before filtering, 153,856 unique peptides were quantified across 28 TMTs. 6841 (4.4%) unique peptides were filtered out due to not being identical in all 8 strains.*

*We have added this to the manuscript.*

Based on the sampling sensitivity of phospho-proteomics, a certain fraction of the phosphorylation sites would be assayed. Can the authors provide estimates, based on their knowledge of MS and genetics how many phosphorylation events may be genetically regulated?

*Reply: We have only reported phosphorylation sites that achieve strong statistical support for genetic regulation. The criteria for genetic association are stringent and there are likely many real genetic effects that don’t clear the bar. Genetic regulation is often a matter of degree and not a black and white distinction. It is an interesting question, but it would difficult to define precisely (quantitively) what it means for a phosphorylation event to be genetically regulated versus not regulated.*

Supplementary Figure 1A - Why are there fewer phosphorylation sites, on average, for heart?

*Reply: 2.4, 2.1, 2.1 phosphorylation sites were identified per protein in heart, kidney and liver tissue, respectively. The reviewer is correct that per protein there were slightly more phosphorylation sites identified in heart tissue. We believe that this may be due to the heart-specific protein, Titan (Ttn), which had 148 phosphopeptides quantified.*

Supplementary figure 1C is missing, and is not called out in the text.

*Reply: This has been corrected.*

How the standardized sex effect is computed is not entirely clear. I'd suggest the authors provide a bit more methodological information describing how the histograms in Figure 2A are generated.

*Reply: The standardized sex effect is computed as the regression coefficient (Y ~ Sex) divided by its standard error.*

*### Minor comments:*

*Page 5 Line 29 - should be "pQTL" here? "distant phQTL (**11% are present in at least two tissues)"*

*Reply: Corrected.*

Page 3 Line 7-8 "i.e.,"

*Reply: Corrected.*

Page 3 Lines 45-46 Would say only slightly correlated, not "correlated", given the R-squared values.

*Reply: Corrected.*

Page 32 Line 4 "(middle), and"

*Reply: Corrected.*

Seems like phQTL is being used for phenotype QTL.

*Reply: We have defined phQTL and adj-phQTL in this manuscript. Prefixes to QTL are often used to represent different classes of (usually molecular) traits. The usage varies somewhat across the literature. In most cases, (clinical) phenotype genetic associations are denoted simply as “QTL”. If it remains a concern, we could modify to “phosQTL”, but given the number of changes that would need be made to the figures, we are content with the current usage.*

**References**

1.	Beausoleil, S.A., Villen, J., Gerber, S.A., Rush, J. & Gygi, S.P. A probability-based approach for high-throughput protein phosphorylation analysis and site localization. *Nat Biotechnol* **24**, 1285-1292 (2006).

2.	Keele, G.R. et al. Regulation of protein abundance in genetically diverse mouse populations. *Cell Genomics* **1**, 100003 (2021).

**Second round of review**

**Reviewer 1**

Overall, I think the authors have addressed my comments, and improved the release of data supporting findings. In an ideal world, all the data uploaded to FigShare would be part of the PX submission, but I realise it can be a pain to resubmit or update PX, so hopefully FigShare will remain long term available.

**Reviewer 2**

The authors have addressed by concerns.
